# The NLRP3-Inflammasome-Caspase-1 Pathway Is Upregulated in Idiopathic Pulmonary Fibrosis and Acute Exacerbations and Is Inducible by Apoptotic A549 Cells

**DOI:** 10.3389/fimmu.2021.642855

**Published:** 2021-04-23

**Authors:** Benedikt Jäger, Benjamin Seeliger, Oliver Terwolbeck, Gregor Warnecke, Tobias Welte, Meike Müller, Christian Bode, Antje Prasse

**Affiliations:** ^1^ Fraunhofer Institute for Toxicology and Experimental Medicine, Hannover, Germany; ^2^ Department of Respiratory Medicine, University Medical Center, Freiburg, Germany; ^3^ Faculty of Biology, Albert Ludwig University, Freiburg, Germany; ^4^ Department of Respiratory Medicine, Hannover Medical School and Biomedical Research in End-stage and Obstructive Lung Disease (BREATH), German Center for Lung Research (DZL), Hannover, Germany; ^5^ Department of Heart, Thoracic, Transplantation and Vascular Surgery, Hannover Medical School, Hannover, Germany; ^6^ Department of Anesthesiology and Intensive Care Medicine, University Hospital Bonn, Bonn, Germany

**Keywords:** idiopathic pulmonary fibrosis, inflammasome, NLRP3, acute exacerbation, inflammation

## Abstract

Idiopathic pulmonary fibrosis (IPF) is a relentlessly progressive disease harboring significant morbidity and mortality despite recent advances in therapy. Regardless of disease severity acute exacerbations (IPF-AEs) may occur leading to considerable loss of function and are the leading cause of death in IPF. Histologic features of IPF-AE are very similar to acute respiratory distress syndrome (ARDS), but the underlying mechanisms are incompletely understood. We investigated the role of the NLRP3 inflammasome in IPF and IPF-AE. Bronchoalveolar lavage (BAL) cells were sampled from patients with IPF (n = 32), IPF-AE (n = 10), ARDS (n = 7) and healthy volunteers (HV, n = 37) and the NLRP3-inflammasome was stimulated *in-vitro*. We found the NLRP3 inflammasome to be hyper-inducible in IPF compared to HV with increased IL-1ß and pro-IL-1ß levels on ELISA upon stimulation as well as increased caspase-1 activity measured by caspase-1p20 immunoblotting. In IPF-AE, IL-1ß was massively elevated to an extent similar to ARDS. To evaluate potential mechanisms, we co-cultured BAL cells with radiated A549 cells (a model to simulate apoptotic alveolar epithelial cells), which led to increased NLRP3 mRNA expression and increased caspase-1 dependent IL-1ß production. In the presence of a reactive oxygen species (ROS) inhibitor (diphenyleneiodonium) and a cathepsin B inhibitor (E64D), NLRP3 expression was suppressed indicating that induction of NLRP3 activation following efferocytosis of apoptotic A549 cells is mediated *via* ROS and cathepsin-B. In summary, we present evidence of involvement of the NLRP3 inflammasome-caspase pathway in the pathogenesis of IPF-AE, similarly to ARDS, which may be mediated by efferocytosis of apoptotic alveolar epithelial cells in IPF.

## Introduction

Idiopathic pulmonary fibrosis (IPF) is the most common of the idiopathic interstitial pneumonias and is characterized by its progressive nature and considerable mortality despite recent advances in antifibrotic therapy (1, 2). A severe complication of IPF may occur in the form of an acute exacerbation (IPF-AE), defined as unexplained worsening of the condition with new bilateral ground glass opacifications on chest computer tomography (CT) without evidence of pulmonary edema or lung embolism, overall sharing many features with acute respiratory distress syndrome (ARDS) (3, 4). Known triggers include lung injury due to thoracic surgery, chest trauma, invasive ventilation but also aspiration and infections (3). Notably, IPF-AE may occur irrespectively of disease severity and harbors a poor prognosis with in-hospital mortality of 50% (3). The most common histopathological pattern found in these patients is diffuse alveolar damage which is also found in ARDS (5, 6). The underlying pathomechanisms of IPF-AE are however, still poorly understood.

The NLRP3-inflammasome has been associated with various pulmonary diseases, including sarcoidosis (7), asbestosis and silicosis (8), rheumatoid arthritis associated interstitial lung disease and also IPF (9), but has not been studied in IPF-AE. Inflammasomes are multiprotein complexes which include a sensor, the adapter protein apoptosis-associated speck-like protein containing a CARD domain (ASC) and caspase-1 (10). Sensors include among others the nucleotide-binding oligomerization domain-like receptor (NLR) family, pyrin domain-containing 3 (NLRP3) (10). For the activation of caspase-1, two signals are necessary: signal 1 induces activation of pattern-recognition receptors (PRR) for example Toll-like receptors (TLRs) by pathogen associated molecular patterns (PAMPS) including lipopolysaccharides (LPS). This leads to nuclear factor kappa-light-chain-enhancer of activated B cells (NF-*κ*B) regulated transcription of inflammasome components: pro-caspase-1, pro–IL-1β and NLRP3 (10). Signal 2 includes a magnitude of stimuli, including ATP and nigericin, which activate NLRP3 inducing inflammasome assembly *via* the oligomerization of ASC. This results in the activation of pro-caspase-1, which is spliced into its active forms caspase-1p10 and caspase 1p20 which in turn activate pro-IL-1β *via* proteolytic cleavage into the active cytokine IL-1β (10, 11). In ARDS, key cytokines include the caspase-1 dependent IL-1ß and IL-18 and the involvement of the NLRP3-inflammasome has been recently demonstrated (12, 13).

IPF pathogenesis is driven by dysfunctional repair mechanisms to microinjuries of the alveolar epithelium and especially of type II alveolar epithelial cells (AECs) (14). In the presence of persistent stress, the AEC-II dysfunctional response lead to cell apoptosis (15). These apoptotic cells are then ingested by alveolar macrophages (AMs), a process which is called efferocytosis (16), capable of providing a pro-fibrotic macrophage response which can induce pulmonary fibrosis (17). Dysregulated efferocytosis observed in IPF (18) can lead to increased production of reactive oxygen species (ROS). Interestingly also activation of the NLRP3 inflammasome by many trigger factors was shown to rely on ROS production (19–25) and cathepsin B leakage into the cytoplasm (26), suggesting a link between efferocytosis in IPF and NLRP3-inflammasome activation.

We aimed to examine the inducibility of the NLRP3-inflammasome in IPF, IPF-AE, and ARDS and the potential role of apoptotic epithelial cells in promoting NLRP3-inflammasome inducibility. We provide evidence of a hyper-inducible NLRP3-inflammasome-caspase 1 pathway which can be triggered by efferocytosis of apoptotic AECs.

## Material and Methods

### Patient Population and Bronchoalveolar Lavage Sample Preparation

BRONCHOALVEOLAR Lavage (BAL) sampling was performed in patients with acute respiratory distress syndrome (ARDS), idiopathic pulmonary fibrosis (IPF) with and without acute exacerbation (IPF-AE), and healthy volunteers (HV) at the University Medical Center in Freiburg im Breisgau (Germany) and at Hannover Medical School (Germany). Healthy volunteers were screened for pulmonary abnormalities by thorough medical history, physical examination, and pulmonary function testing. Diagnosis of ARDS was made in accordance with the Berlin 2012 definition (4), and a diagnosis of IPF was established per the practice guidelines issued by the American Thoracic Society and European Respiratory Society (1). The definition criteria of AE were: deterioration of dyspnoea >20% in <3 weeks, occurrence of new opacities, and absence of alternative cause (including infection, heart failure, or pulmonary embolism) (27). All IPF patients with suspicion of acute exacerbation received chest computed tomography.

All patients provided written informed consent, and collection of bio-samples was registered at the German Clinical Trials Register (DRKS00000017 and DRKS00000620). The respective institutional review boards approved of the bio-sampling (Freiburg 47/06 March 10^th^ 2006, Hannover, #2923-2015 and #2516-2014, Nov 2^nd^ 2015).

BAL was performed in the middle lobe or lingula. After BAL sampling, macro-impurities were removed by sample filtration through sterile gauze. Differential BAL total cell number was counted using May–Grunwald–Giemsa stain (Merck) on native sample. BAL samples were centrifuged at 500g for 10min at 4°C (28) and subsequently resuspended in Macrophage SFM Medium or DMEM medium. BALs were performed between 2010 and 2013 at the University Medical Center Freiburg and in 2017 and 2021 at Hannover Medical School.

### Cell-Culture and NLRP3-Inflammasome Stimulation Protocol

**Figure 1:**
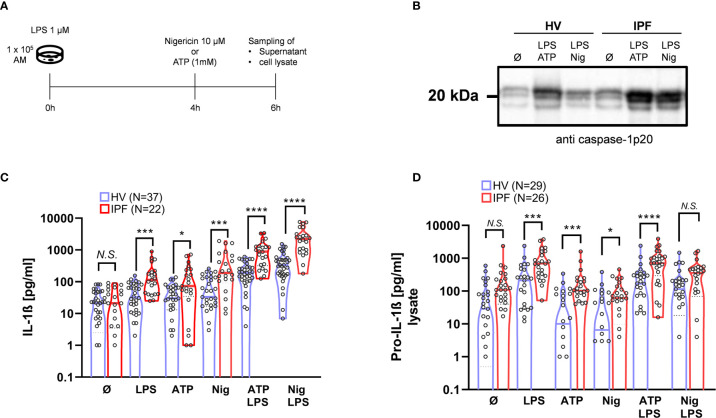
The ability to activate the NLRP3 inflammasome is significantly increased in BAL cells from IPF patients. For NLRP3 stimulation, bronchoalveolar lavage (BAL) cells were incubated and stimulated with LPS at 0h, following Nigericin or ATP stimulation after 4h **(A)**. In both healthy volunteers (HVs) and idiopathic pulmonary fibrosis (IPF) there was a significant increase in IL-1ß production following NLRP3-inflammasome stimulation protocol **(B)**, with significant higher responses in IPF compared to HV. Caspase-1 activation is demonstrated by representative immunoblotting of cleaved caspase-1p20 (representative blot shown; total of n = 15 immunoblots performed). **(C)**. Pro-IL-1ß concentration in cell lysates was elevated in IPF patients compared to HV following stimulation with LPS, ATP, Nigericin, and LPS + ATP **(D)**. IL-1ß and Pro-IL-1ß values were compared using unpaired t-tests; *p < 0.05; ***p < 0.001; ****p < 0.0001, *N.S*. non-significant.

The NLRP3 inflammasome was activated in accordance to our recently described protocol (7). In brief, 1 × 10^5^ BAL cells derived from HV, IPF, and ARDS patients, were incubated with 100 µl of Macrophage SFM/Gibco Medium (Life Technologies, USA) with 1% penicillin and streptomycin (Biochrom, Germany) at 37°C and 5% CO_2_ in a flat-bottom 96 well plate (**Figure 1A**). Two steps are needed for the activation of inflammasome-dependent caspase-1 activation: a first signal 1 such as an Toll-like-receptor agonist (LPS) and a second tissue damage signal such as ATP or Nigericin (29). BAL-cells were primed with 1 µM LPS (Fluka Biochemika, Switzerland) initially for 4h. Thereafter, 1 mM ATP (Sigma Aldrich, USA) or 10 µM Nigericin (Sigma Aldrich, USA) was added with incubation of another 2h. Supernatants were sampled and stored at −80°C until analysis. Cells were lysed with lysis buffer [PBS (Life Technologies, USA) with 0.5% triton X (Sigma Aldrich, USA) and 10% FCS (Biochrom, Germany)] for pro-IL-1ß ELISA and with 200 µl Trizol (Thermo Fisher Scientific, USA) for RNA isolation and stored at −80°C until analysis.

### Cell Culture and Stimulation With Co-Culturing BAL Cells With Radiated A549 Cells

Since chronic cellular stress has been demonstrated to induce AEC apoptosis and consequently efferocytosis by alveolar macrophages, which is believed to contribute to IPF pathogenesis, we examined the effects of co-culturing alveolar macrophages with apoptotic A549 cells on NLRP3-inflammasome activation (15, 16). A549 cells were cultured in DMEM/Gibco medium (Life Technologies, USA) with 10% FCS and 1% penicillin/streptomycin at 37°C/5% CO_2_ and radiated with 10 Gy and then incubated for additional 72h (30–32). Harvesting after 72h resulted in highest rate of apoptotic cells on viability testing. Viability was assessed *via* trypan blue staining and the FACS annexin V apoptosis detection Kit (BD, USA) as per manufacturer’s instructions. 1 × 10^6^ BAL-cells were then co-cultured w/wo 1 × 10^5^ radiated A549 cells in 500 µl of macrophage/Gibco medium (serum free) in a 24-well cell culture plate and incubated for 1h at 37°C/5% CO_2_ to allow for efferocytosis and consequently stimulated as described above.

To evaluate the effects of efferocytosis on NLRP3 mRNA expression, we co-cultured radiated, apoptotic A459 with normal BAL-cells for 1h. Following that, we stimulated BAL cells with 1 µM LPS in a 24-well cell culture plate for 2h at 37°C. Supernatants were discarded, and cells were lysed with 200 µl Trizol. In some experiments, we assessed the effect of the NADPH-Oxidase inhibitor [ROS-inhibitor; DPI; 20 µM (Sigma Aldrich, USA)] and of the cathepsin B inhibitor E64D (10 µM) (MCE, USA). Both compounds were added with the radiated A549 cells at baseline, and DMSO 0.2% was added as vehicle (both E64D and DPI were suspended in DMSO). Cell lysates were again sampled in 200 µl Trizol following 2h incubation at 37°C/5% CO_2._ In another set of experiments, the effects of the selective NLRP3 inhibitor (MCC950) (33) and caspase-1 inhibitor VX-765 (both Invivogen, France) on IL-1ß and TNF-α production following inflammasome stimulation were evaluated. Two different concentrations (1 + 10 µM and 25 + 75 µM, respectively) were added 30min before addition of radiated A549 cells.

### Efferocytosis Assay

To demonstrate efferocytosis of radiated A549 cells by alveolar macrophages, 1 × 10^5^ control BAL cells were incubated with 2 µM cytochalasin D (Sigma Aldrich #250255, USA), a known cytoskeletal disruptor of phagocytosis in macrophages, for 30min at 37°C. Radiated A549 cells were incubated with pHrodo (Red AM Intracellular pH Indicator, Thermo Fisher Scientific #P35372, USA) at a 1:100,000 dilution for 30min. Engulfment of A549 cells leads to a pH-shift in the alveolar macrophages inducing an increased pHrodo light emission (34). Afterwards 1 × 10^5^ BAL cells were incubated with and without 3 × 10^5^ pHrodo-labeled radiated A549 cells for 2h in a 96 well plate. All BAL cells with and without A549 cells were harvested with 100 µl PBS, and nuclear staining was performed with 1 µg/ml Hoechst-33342 dye solution (Thermo Fisher Scientific #H3570, USA) for 30min at RT. Immediately afterwards, a cytospin with 2 × 10^4^ cells in 100 µl PBS was performed, and efferocytosis of macrophages was analyzed by fluorescence microscopy using Axio Observer Inverted microscope and ZEN navigation software (Zeiss, Germany). Percentage of pHrodo^+^ and Hoechst-33342^+^ cells was counted using ImageJ V.1.53e (NIH, USA) (35).

### Immunoblot Analysis of Caspase-1p20 Activity

Supernatants collected after NLRP3-stimulation protocol were precipitated with methanol/chloroform. After initial centrifugation, the upper phase was discarded and methanol was added to the lower phase, followed by another centrifugation step. The supernatant was discarded, and the pellet was incubated at 55°C for 5min and resuspended in 20 µl Laemmli-buffer (Bio-Rad, USA). Samples were consequently cooked for 5min at 95°C. Samples were separated using 15% SDS-PAGE gels and transferred to polyvinylidene difluoride membranes. Cleaved caspase-1 (p20) was detected using primary antibody rabbit mAb cleaved caspase-1 (Asp297) (Cell Signaling Technology, USA) with the secondary antibody goat anti-rabbit (H + L) HRP conjugate (1:3,000) (Bio-Rad, USA). Enhanced chemiluminescence (Clarity™ Western ECL Substrate, Bio-Rad, USA) was used for detection with ChemiDoc™ MD Imaging System (BioRad, USA).

### IL-1ß, pro-IL-1ß and TNF-α Measurement by ELISA

The concentration of both pro-IL-1ß and IL-1β was measured by ELISA (Human IL-1β/IL-1F2 DuoSet, R&D, USA) in the cell lysate and culture supernatant, respectively. (36). TNF-α concentration from culture supernatant was measured by ELISA (Human TNF-alpha DuoSet #DY210, R&D, USA).

### RT-PCR for NLRP3

RNA was isolated *via* Trizol method (ThermoFisher Scientific, USA). 1 × 10^6^ cells were lysed with 200 µl of Trizol. Extracted RNA was reverse-transcribed to cDNA using the iScript cDNA Synthesis kit (Bio-Rad, USA) as per manufacturer’s instructions (7). The obtained cDNA was analyzed by Real-Time PCR (Light Cycler/Roche, Switzerland) with the following primers: huNLRP3 (5′-AGAATGCCTTGGGAGACTCA-3′ and 5′-CAGAATTCACCAACCCCAGT-3′), resulting in a 93 bp product, exon 6/7 overlapping; GAPDH (5′-ACAGTCAGCCGCATCTTCTT-3′ and 5′-GTTAAAAGCAGCCCTGGTGA-3′) as reference (7). The expression of huNLRP3 was normalized to GAPDH expression. A cycle threshold value was calculated and used to ascertain the relative level of huNLRP3 messenger RNA by the following formula: relative expression = [2 (cycle threshold of glyceraldehyde 3-phosphate dehydrogenase- ycle threshold of huNLRP3)] × 10,000 for each complementary DNA sample.

### Statistical Analysis

Statistical analyses were performed using GraphPad Prism 9 Software (La Jolla, USA) and RStudio version 1.3.1093 (RStudio Inc., USA). Variables were compared by unpaired t-test. All data are expressed as mean + SD unless stated otherwise. A two-tailed p-value of <0.05 was considered to statistically significant.

## Results

### Study Population

A total of 86 BALs were used in this study from patients with IPF (n = 32), IPF/AE (n = 10), ARDS (n = 7), and HV (n = 37) with demographics, pulmonary function tests (in IPF), and BAL cell counts shown in **Table 1**. All patients were naïve to antifibrotic therapy, since the majority of patients were recruited prior to the widespread introduction of antifibrotics. At the time of BAL, 41% of IPF and 60% of IPF-AE patients were receiving triple-therapy with n-acetylcystein, prednisolone, and azathioprine. In 2021, additional BALs in three IPF patients were performed (two female; mean age 71 years, mean forced vital capacity 70% of predicted, mean diffusion capacity for carbon monoxide 52% of predicted; mean alveolar macrophages in BAL 87%, mean neutrophils in BAL 4%).

**Table 1:** Study population and BAL differential cell counts.

Characteristics	IPF (n = 32)	IPF/AE (n = 10)	ARDS (n = 7)	HV (n = 37)
Age, years (SD)	68 ± 10	68 ± 10	52 ± 15	29 ± 8
Sex (male), N (%)	30 (94)	9 (90)	6 (86)	20 (54)
Forced vital capacity, % of predicted, mean (SD)	64 ± 16	–	–	–
Diffusion capacity for carbon monoxide (single breath), % of predicted, mean (SD)	36 ± 20	–	–	–
Invasive ventilation, n (%)	0	2 (20)	7 (100)	0
Received n-acetylcysteine, prednisolone, and azathioprine	13 (41)	6 (60)	0	0
BAL cell count, × 10^6^/100ml (SD)	14.6 ± 7.8	18.0 ± 7.5	32.4 ± 30.7	7.0 ± 3.2
Alveolar macrophages, % (SD)	65.1 ± 21.2	59.1 ± 15.5	32.7 ± 19.5	86.0 ± 5.2
Lymphocytes, % (SD)	12.1 ± 12.8	4.0 ± 3.0	8.5 ± 3.6	10.5 ± 5.2
Neutrophils, % (SD)	18.9 ± 22.0	30.8 ± 17.3	56.2 ± 20.9	2.3 ± 1.8
Eosinophils, % (SD)	3.4 ± 4.7	6.3 ± 4.4	2.0 ± 3.9	0.3 ± 0.7

ARDS, acute respiratory distress syndrome; BAL, bronchoalveolar lavage; HVs, healthy volunteers; IPF, idiopathic pulmonary fibrosis; IPF/AE, acute exacerbation of idiopathic pulmonary fibrosis.

### The NLRP3 Inflammasome Activation Is Significantly Increased in AM From IPF Patients Compared to HV

In a first step, the IL-1ß production of normal BAL cells derived from HV was tested following NLRP3-inflammasome stimulation (**Figure 1A**). Normal BAL cells did not spontaneously produce IL-1ß, which production is tightly regulated (37). Mean basal IL-1ß production without stimulation was increased with LPS alone (p = 0.002), Nigericin alone (p = 0.0002), and with ATP alone (p = 0.0005) (**Figure 1B**). Most pronounced IL-1ß production was seen with co-stimulation of LPS + ATP (p < 0.0001) and LPS + Nigericin (p < 0.0001). Evidence of caspase-1 activation by NLRP3 inflammasome was seen by increased protein band intensity of the caspase-1p20 fragment by immunoblot with stimulation by LPS + ATP and LPS + Nigericin (**Figure 1C**).

Compared to BAL cells from IPF patients, basal (unstimulated) IL-1ß production was similar compared to HV. Of note, there was a consistently higher IL-1ß production in BAL cells from IPF patients after stimulation with LPS (p = 0.0002), Nigericin: (p = 0.0001), and ATP (p = 0.0002). With the combined stimulation with LPS + ATP and LPS + Nigericin, discrepancies were even more pronounced (both p < 0.0001).

In line with this, there was a significant increase in caspase-1 activation determined by caspase-1p20 protein-band intensity after co-stimulations in IPF patients compared to HV (**Figure 1C**).

### Intracellular Pro-IL-1ß Protein Expression Following Stimulation Is Increased in BAL-Cells From IPF Patients Compared to HV, but Not Without Stimulation

In order to test whether the NLRP3 inflammasome is already primed (signal 1 activated) in BAL cells derived from IPF patients, we analyzed intracellular pro-IL-1ß levels. Notably, baseline (unstimulated) pro-IL-1ß levels were not statistically significantly elevated in IPF compared to HV, indicating that NLRP3 inflammasome is rather hyper-inducible than primed. As expected, intracellular pro-IL-1ß levels increased following priming with LPS; LPS + ATP and LPS + Nigericin, but not with ATP and Nigericin alone (**Figure 1D**). Compared to HV, pro-IL-1ß production was significantly increased following stimulation with LPS + ATP (p < 0.0001) and LPS alone (p = 0.0003), as well as ATP (p = 0.03) and Nigericin (p = 0.005) in IPF (**Figure 1D**).

### NLRP3 Inflammasome Is Markedly Upregulated in Patients With Acute Exacerbation of IPF to Similar Extent as Patients With Primary ARDS

**Figure 2:**
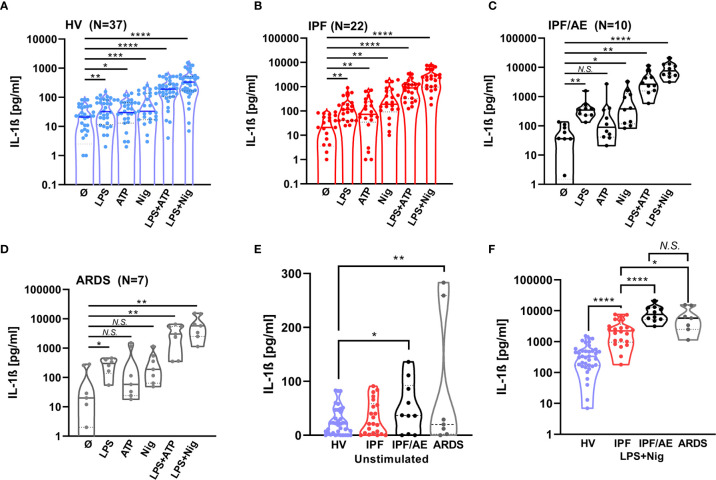
IL-1β production after NLRP3 inflammasome stimulation is highest in BAL cells from patients with an acute exacerbation during IPF and BAL cells from patients with ARDS. IL-1ß production was detected by ELISA and is inducible by NLRP3 stimulation in alveolar macrophages of patients with acute exacerbation of idiopathic pulmonary fibrosis **(C)** and acute respiratory distress syndrome **(D)**. IL-1ß values for healthy volunteers (HVs) and IPF are shown for comparison **(A, B)**. IL-1ß production in these two cohorts is significantly increased compared to healthy volunteers (HVs) to a similar extent at baseline and following stimulation **(E, F)**. IL-1ß levels with stimulation compared to baseline and unstimulated IL-1ß levels between groups and LPS/Nigericin stimulation were compared using unpaired t-tests; *p < 0.05; **p < 0.01; *** p < 0.001; ****p < 0.0001, *N.S.* non-significant.

To see how the NLRP3 inflammasome contributes to IPF/AE, we stimulated BAL cells from patients with IPF/AE (n = 10) with the same protocol as described above. As shown in **Figure 2**, there was a markedly elevated IL-1ß production of BAL cells from patients with both IPF/AE and ARDS following singular and combined stimulation and without stimulation (compared to HV). With IPF/AE and ARDS, the fold-change for IL-1ß production compared to IPF (without AE) for the combined stimuli was 3–4 and 15–20 compared to HV (**Figure 2F**).

### Co-Culturing BAL Cells From HV With Radiated A549 Cells Results in Efferocytosis and Increases NLRP3-Inflammasome Activation. Selective NLRP3 and Caspase-1 Inhibition Suppresses IL-1ß Production Following Co-Incubation

**Figure 3:**
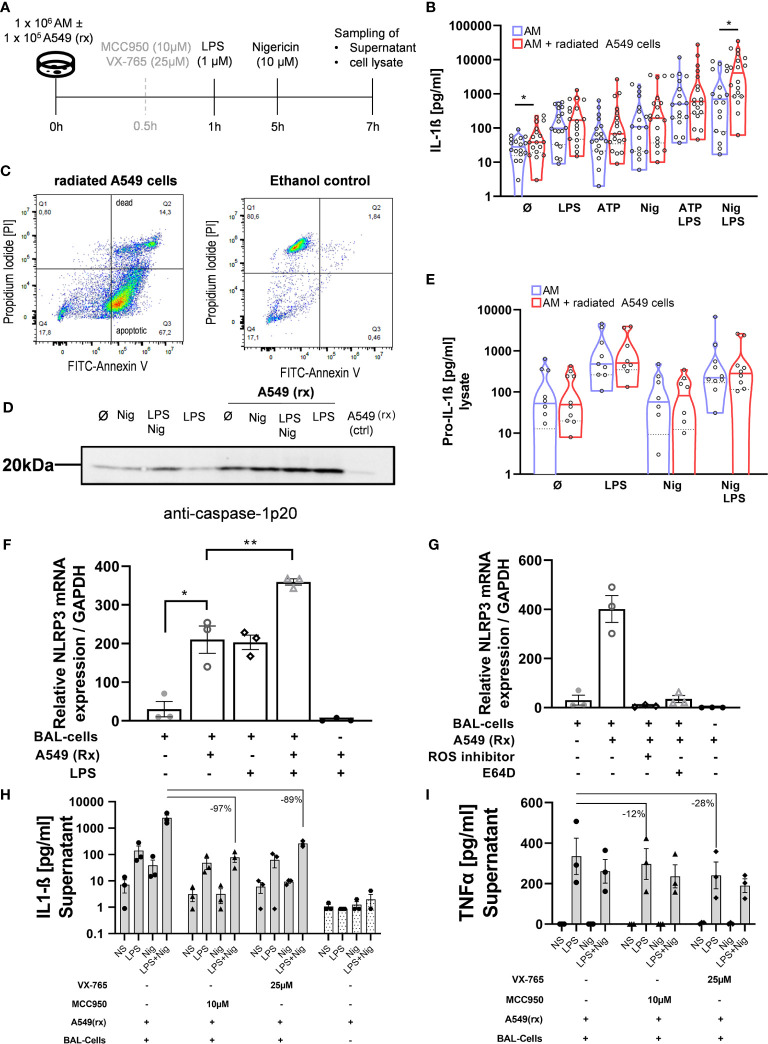
Efferocytosis of apoptotic alveolar epithelial cells activates the NLRP3 inflammasome in AM. BAL cells were co-cultured with the radiated (rx) A549 cells, and the NLRP3 inflammasome was additionally stimulated **(A)**. A549 cells were radiated with 10 Gy and incubated for 72h, following which the majority of the cells were apoptotic demonstrated by Annexin-V staining **(C)**. There were increased IL-1ß levels with all stimulations, which were statistically significant at baseline and following LPS + Nigericin (Nig) stimulation **(B)**. Increased caspase-1 activation with A549(rx) co-culture was demonstrated by immunoblotting of cleaved caspase-1p20 subsegment (representative blot shown; total of n = 3 immunoblots performed) **(D)**. Pro-IL-1ß levels in cell lysate were not different between BAL cells and BAL cells co-cultured with A549(rx) cells **(E)**. NLRP3 mRNA expression was assessed after 2h of stimulation, w/wo the presence of A549(rx). BAL cells cocultured with A459(rx) expressed NLRP3-mRNA in a similar range as BAL cells stimulated with LPS alone **(F)**. Combined stimulation of LPS and A549(rx) resulted in a marked increase in NLRP3-mRNA expression. The effect on NRLP3-mRNA expression (relative to GAPDH) by efferocytosis was inhibited by either a NADPH-Oxidase inhibitor (ROS-inhibitor; DPI) or a cathepsin inhibitor (E64D) **(G)**. IL-1ß production could be inhibited by inhibition of NLRP3 (MCC950) and also caspase-1 (VX-765) **(H)**, while TNF-α levels were largely retained **(I)**. IL-1ß levels and NLRP3 mRNA levels were compared using unpaired t-tests; *p < 0.05; **p < 0.01; *N.S.* non-significant.

**Figure 4:**
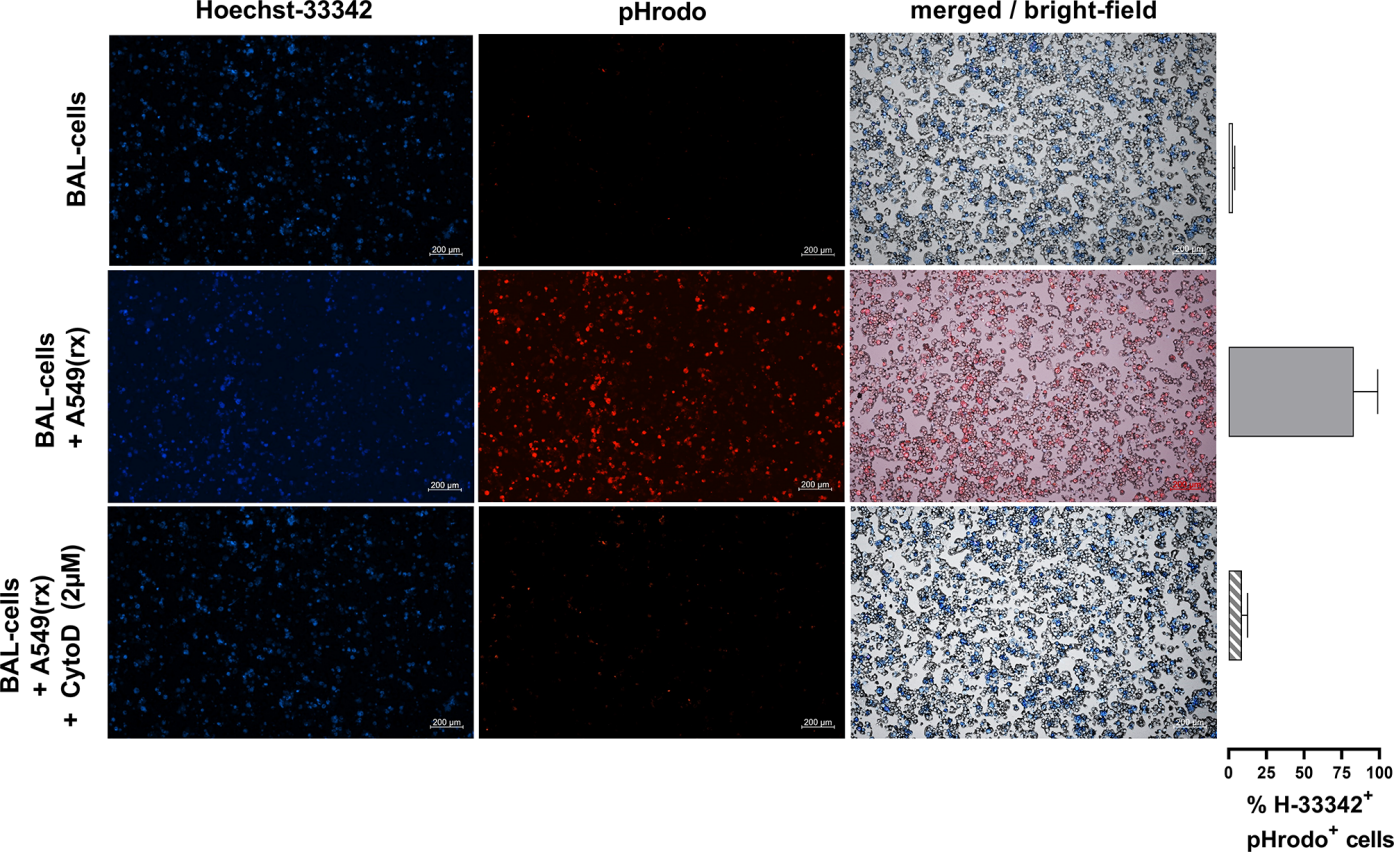
Co-incubation of alveolar macrophages with pHrodo-labeled radiated A549 cells leads to efferocytosis of A549 cells. Nucleolar staining with Hoechst-33342 of BAL cells was performed. Following co-incubation with pHrodo labeled radiated A549 cells, pHrodo positive alveolar macrophages were detected, indicating efferocytosis of A549 cells. Following pre-treatment with cytochalasin-D (2 µM), this effect was almost completely inhibited. Exemplary fluorescence microscopy images (5× magnification) of BAL cells with and without co-incubation and pre-treatment of cytochalasin D are shown, and the percentages of Hoechst^+^/pHrodo^+^ cells of all Hoechst^+^ cells are shown in the right hand panel. Bronchoalveolar lavages from three patients were used and measured in triplicates for each condition.

To further investigate potential mechanisms behind the increased NLRP3 inflammasome activation in IPF patients *vs* HV, AECs (A549) were radiated with 10 Gy and harvested 72h after radiation (**Figure 3A**). Radiation induced apoptosis in the majority of cells, while a small proportion was double positive for annexin V and propidium iodide (**Figure 3C**). To verify if efferocytosis was actually performed, we labeled radiated A549 cells with pHrodo before co-incubation with BAL cells. Following co-incubation with A549 cells, alveolar macrophages became pHrodo^+^ in 83% of all Hoechst-33342^+^ cells, indicating efferocytosis of A549 cells. Following pre-treatment with cytochalasin-D (2 µM), this effect was almost completely inhibited (9% pHrodo^+^/H-33342^+^ cells) (**Figure 4**).

BAL cells of HV were co-incubated with radiated A549 cells for 1h before running the NLRP3 stimulation protocol. There was a significant 2.2-fold increase in IL-1ß production in BAL cells co-cultured with radiated A549 at baseline (without stimulation; p = 0.031) and following stimulation with LPS + Nigericin (3.5-fold; p = 0.025) (**Figure 3B**). With the other stimulations, IL-1ß production was increased but did not reach statistical significance. Equally, on immunoblotting there was an increased protein band both with caspase 1-p20 with co-culturing of radiated A549 (**Figure 3D**). There was no difference in pro-IL-1ß concentration in the cell lysates with or without co-culturing of A549 (**Figure 3E**).

To evaluate if the IL-1ß response was mainly mediated *via* the NLRP3 inflammasome, we pre-treated BAL cells with a selective NLRP3-inhibitor (MCC-950) or caspase-1 inhibitor (VX-765) before co-culturing the A549 cells (**Figure 3A**). Both agents suppressed IL-1ß production >90% (**Figure 3H**), while TNF-α production remained largely (**Figure 3I**). These results indicate the increased IL-1ß response following efferocytosis of A549 cells is primarily mediated *via* the NLRP3/caspase-1 pathway.

### NLRP3-Gene Expression Is Increased by Co-Culturing With Apoptotic A549 Cells and Attenuated by Inhibition of ROS and Cathepsin B

Acute lung injury and consecutive epithelial cell death trigger AE-IPF and ARDS. On this background we got interested in the role of efferocytosis in NLRP3 inflammasome activation. We therefore induced apoptosis of the AEC line A549 using radiation. Indeed, 72h after radiation 67% of A549 cells were apoptotic and only 14% necrotic (**Figure 3C**). These apoptotic A549 cells were then co-cultured with normal BAL cells for 1h and resulted in their phagocytosis by AM (**Figure 4**). Co-culturing with radiated A549 cells induced NLRP3 mRNA expression relative to GAPDH in a similar magnitude compared to stimulation with LPS and was significantly increased compared to baseline NLRP3 expression (**Figure 3F**). Combined stimulation with radiated A549 and LPS induced high NLRP3 gene expression. To evaluate possible mechanisms for the increased NLRP3 mRNA expression *via* radiated A549 cells, we tested a ROS and cathepsin B inhibitor. In the presence of a ROS inhibitor (DPI) or a cathepsin B inhibitor (E64D), the NRLP3 mRNA expression in response to co-culturing with radiated A549 cells was completely suppressed (**Figure 3G**), while GAPDH expression was maintained.

## Discussion

Although IPF-AE is the leading cause of death in IPF, underlying mechanisms are poorly understood (38). In this study we demonstrate that the NLRP3-inflammasome is hyper-inducible in BAL cells from IPF patients compared to HV. BAL cells harvested during IPF-AE produced extraordinarily high IL-1ß levels in a similar range as BAL cells of ARDS patients. We found that one potential mechanism driving NLRP3 hyperactivation in IPF-AE may be efferocytosis of apoptotic cells which led to increased NLRP3 expression and caspase-1 activation. The IL-1ß response could be almost completely suppressed by specific inhibition of NLRP3 and caspase-1-activity. Inhibition of ROS or cathepsin B signaling blocked the effect of co-culturing with radiated A549 cells on NLRP3-mRNA transcription in BAL cells.

Our data indicate that the NLRP3 inflammasome is hyper-inducible in IPF BAL cells and especially during acute exacerbation. BAL cells harvested at the time of IPF-AE produced tremendously high levels of IL-1ß and in a similar range as AMs of patients with ARDS. While the role and significant involvement of the NLRP3-inflammasome in ARDS are well documented, activation of the NLRP3 inflammasome pathway in IPF-AE is a novel finding. Our observations in BAL cells from IPF patients are in line with previous observations of elevated IL-1ß and IL-1ß mRNA in IPF (10, 35–37). Activation of the NLRP3-inflammasome pathway has been demonstrated to induce IL-1ß and IL-18 production of BAL cells and is tightly regulated (11). Although we have not purified macrophages from BAL prior to inflammasome stimulation protocol and therefore cannot exclude contribution of other cell types, we think, in line with the literature, that our experiments mainly reflect inflammasome activation of alveolar macrophages. In addition, the relatively short incubation time of 6h used in this study render cell–cell contact dependent mechanisms unlikely as the primary driver for increased NLRP3 activation and IL-1ß production. Furthermore, the neutrophil count between IPF patients with and without AE was relatively small owing to inclusion of IPF patients with advanced disease (39), while the changes in NLRP3-inflammasome inducibility between these groups were considerable.

Several factors have been shown to activate the NLRP3 inflammasome such as phagocytosis of crystalline substances such as silicates or cholesterol but is also triggered by viral and bacterial infection releasing LPS and other TLR ligands as well as DNA and RNA (12, 40). Of note, mechanical ventilation and ventilation induced lung injury also activated the NLRP3-inflammasome in murine models (41, 42). These triggers are also commonly reported to precede acute exacerbation in IPF (3, 27), and it is thus conceivable that elevated inflammasome inducibility in IPF patients (such as the increased pro-IL-1ß an IL-1ß production shown herein upon signal 1 or signal 2 stimulation) predisposes them to increased risk of acute exacerbation when exposed to these stimuli. Notably, in a multicentric BAL gene expression analysis in IPF, a gene set was identified to carry poor prognosis, among which the IL-1ß gene and the NLRP3 gene were included (43). Thus, our data indicate a role of NLRP3 inflammasome signaling in acute exacerbation of IPF.

The NLRP3 inflammasome and IL-1ß signaling are closely linked to neutrophil influx and represent a key pathway in response to cell injury (44–46). IL-1ß and the NLRP3 inflammasome activation also are part of an acute injury response and promote fibrosis and pro-fibrotic pathways such as transforming growth factor (TGF)-*ß* signaling in animal models (47–50). Inflammation caused by bleomycin induced lung injury is triggered by NLRP3 inflammasome activation, while the resulting inflammation and fibrosis were completely prevented by NLRP3 inhibitors (48). Moreover, asbestos and silica, which are known inducers of pulmonary fibrosis, are capable of activating the NLRP3 inflammasome with subsequent IL-1ß production (51). IL-1ß itself can promote TGF-*ß* responses, considered as a key driver in pulmonary fibrosis *via* fibroblast activation (52). Interestingly, both approved antifibrotic agents for the treatment of IPF (nintedanib and pirfenidone) have been shown to reduce IL-1ß expression in lung tissues (53, 54), which might be a mechanism for the reduced risk of acute exacerbation in patients receiving antifibrotic treatment (55, 56).

Another key finding of our study is that co-incubation of BAL cells with apoptotic AEC (A549) and their phagocytosis increase inflammasome-dependent caspase-1 activation. Our model for inducing apoptosis in A549 AECs is well accepted (30–32), and we demonstrated apoptosis in the majority of cells *via* annexin-V expression, and we also demonstrate their efferocytosis by alveolar macrophages. Phagocytosis and generation of phagolysosomes are linked to ROS production and lysosomal stress. Interestingly also activation of the NLRP3 inflammasome by many trigger factors was shown to rely on ROS production (19–25) and cathepsin B leakage into the cytoplasm (26). Based on these findings we tested the effects of ROS or cathepsin B inhibition and found that both of them completely attenuated NLRP3 expression in the context of efferocytosis (16). Studies suggest that ROS, among other direct effects on inflammasome assembly, also upregulates NLRP3 gene expression *via* TLR-4 signaling (57), providing a potential mechanisms for the reduction in NLRP3-mRNA expression observed in this study. A recent murine model showed that apoptotic AEC-II induce a pro-fibrotic gene expression signature in AM following efferocytosis, which on repeated exposure can induce pulmonary fibrosis (17), a process which may be equally important in IPF. Previous animal studies have also implicated that impaired autophagy mechanisms in IPF and aging result in deranged mitochondrial turnover resulting in mitochondria-generated reactive oxygen species (ROS) leading to caspase-1 activation (19–25, 50). Equally, recent reports demonstrated a dependency on cathepsin B (which is also induced by nigericin used in our model) for NLRP3-inflammasome assembly (58, 59). Cathepsin B is released into the cytoplasm upon lysosomal membrane permeabilization (26). Thus, efferocytosis triggers NLRP3 inflammasome activation in alveolar macrophages. While ROS inhibitors convey multifactorial effects, the possibility to suppress the observed IL-1ß response by the specific NLRP3-inhibitor MCC950 (33) carries potential treatment options for IPF-AE and other forms of acute respiratory failure such as COVID-19 (60), which remain to be clinically studied.

Our study has significant limitations to consider: Our data derived from human samples demonstrates evidence for an increased activation of the NLRP3 inflammasome, but given a limited number of measured inflammasome components, we cannot exclude that additional inflammasome subtypes may have equally been activated. Age, comorbidities, and respiratory failure in patients with IPF-AE often preclude these patients from undergoing bronchoscopy which primary function lies in exclusion of infection, rendering these bio samples available only in few cases recruited in the era before antifibrotic treatment was available. The considerable younger age of the recruited healthy volunteers also limits comparability to IPF patients since inflammasome activation may also occur with aging alone (61). Although the differences observed in the present study appear unlikely to be explained solely by age, at least a contributory effect can be assumed. Another important limitation of this study is that we did not purify macrophages prior to the described *in vitro* experiments. On one side this experimental protocol leaves cells untouched since methods for cell purification have been shown to activate macrophages. On the other hand, because we studied BAL cells in complete, we cannot exclude a major contribution of other cell types such as neutrophils (62). Neutrophils have been recently shown to activate NLRP3 inflammasome upon respiratory infections (63) which can be achieved *via* production of neutrophil extracellular traps (44, 45). Compared with macrophages, neutrophils are however short-lived and difficult to study *in vitro* because most of the neutrophils are lost within 2h of cell culture.

In conclusion, our study demonstrates in human BAL cells the hyper-inducibility of the NLRP3-inflammasome in IPF and particularly at acute exacerbation. We identified efferocytosis of apoptotic cells caused by lung injury as one responsible mechanism. The activated NLRP3 inflammasome in IPF-AE may be of potential therapeutic value, and compounds blocking NLRP3-inflammasome activation might be of potential benefit for this fatal condition (53). Future research is needed to explore the clinical role of inflammasome inhibitors on the course of pulmonary fibrosis and acute exacerbation in particular.

## Data Availability Statement

The raw data supporting the conclusions of this article will be made available by the authors without undue reservation.

## Ethics Statement

The studies involving human participants were reviewed and approved by the Ethics committee of The University of Freiburg im Breisgau. The patients/participants provided their written informed consent to participate in this study.

## Author Contributions

BJ and BS wrote the first draft of the manuscript, conducted the experiments, contributed to the study design, and interpreted the data. OT conducted the experiments, provided technical support, and revised the manuscript. GW, TW, and CB interpreted the data, critically revised the manuscript, and supervised the project. MM conducted the experiments, provided technical support, and interpreted the data. AP designed the study, supervised the project, interpreted the data, and critically revised the manuscript. All authors contributed to the article and approved the submitted version. All authors take responsibility for the integrity of the data.

## Funding

BS is supported by PRACTIS–Clinician Scientist Programme of Hannover Medical School, funded by the Deutsche Forschungsgemeinschaft (DFG, ME 3696/3-1) and the German Center for Lung Research (DZL). BS, TW, and AP are supported by KFO311. AP is supported by E-RARE IPF-AE.

## Conflict of Interest

The authors declare that the research was conducted in the absence of any commercial or financial relationships that could be construed as a potential conflict of interest.
